# Effects of Oral Administration of Chitin Nanofiber on Plasma Metabolites and Gut Microorganisms

**DOI:** 10.3390/ijms160921931

**Published:** 2015-09-10

**Authors:** Kazuo Azuma, Ryotaro Izumi, Mari Kawata, Tomone Nagae, Tomohiro Osaki, Yusuke Murahata, Takeshi Tsuka, Tomohiro Imagawa, Norihiko Ito, Yoshiharu Okamoto, Minoru Morimoto, Hironori Izawa, Hiroyuki Saimoto, Shinsuke Ifuku

**Affiliations:** 1 Department of Clinical Veterinary Medicine, Faculty of Agriculture, Tottori University, Tottori 680-8533, Japan; E-Mails: tosaki@muses.tottori-u.ac.jp (T.O.); ymurahata@muses.tottori-u.ac.jp (Y.M.); tsuka@muses.tottori-u.ac.jp (T.T.); imagawat@muses.tottori-u.ac.jp (T.I.); taro@muses.tottori-u.ac.jp (N.I.); yokamoto@muses.tottori-u.ac.jp (Y.O.); 2 Graduate School of Engineering, Tottori University, Tottori 680-8552, Japan; E-Mails: jastam0ment@gmail.com (R.I.); o_o_mari11@yahoo.co.jp (M.K.); tomonekomame@gmail.com (T.N.); h-izawa@chem.tottori-u.ac.jp (H.I.); saimoto@chem.tottori-u.ac.jp (H.S.); 3 Division of Instrumental Analysis, Research Center for Bioscience and Technology, Tottori University, Tottori 680-8550, Japan; E-Mail: morimoro@chem.tottori-u.ac.jp

**Keywords:** chitin nanofibers, surface-deacetylated chitin nanofibers, metabolome analysis, ATP, serotonin, acyl-carnitine, fatty acids

## Abstract

The aim of this study was to examine the effects of oral administration of chitin nanofibers (CNFs) and surface-deacetylated (SDA) CNFs on plasma metabolites using metabolome analysis. Furthermore, we determined the changes in gut microbiota and fecal organic acid concentrations following oral administrations of CNFs and SDACNFs. Healthy female mice (six-week-old) were fed a normal diet and administered tap water with 0.1% (*v*/*v*) CNFs or SDACNFs for 28 days. Oral administration of CNFs increased plasma levels of adenosine triphosphate (ATP), adenosine diphosphate (ADP), and serotonin (5-hydroxytryptamine, 5-HT). Oral administration of SDACNFs affected the metabolisms of acyl-carnitines and fatty acids. The fecal organic level analysis indicated that oral administration of CNFs stimulated and activated the functions of microbiota. These results indicate that oral administration of CNFs increases plasma levels of ATP and 5-HT via activation of gut microbiota.

## 1. Introduction

Chitin (β-(1-4)-poly-*N*-acetyl-d-glucosamine) is widely distributed in nature and is the second most abundant polysaccharide after cellulose [1]. It occurs in nature as ordered macrofibrils and is the major structural component of the exoskeleton of crab and shrimp shells and the cell wall of fungi and yeast [2]. Recently, simple methods for the preparation of chitin nanofibers (CNFs) were reported [3,4,5]. In addition, the preparation of surface-deacetylated CNFs (SDACNFs) has been reported by Isogai *et al.* [6]. This study showed that while the nanofiber surface was transformed into chitosan by deacetylation, the core was maintained as chitin crystal. The cationic electrostatic repulsive force facilitated the easy disintegration of the deacetylated chitin into nanofibers, and the SDACNFs were homogeneously dispersed in water. These nanofibers are considered to have great potential for various applications because they have several useful properties such as high specific surface area and high porosity [2].

Previously, CNFs and SDACNFs were reported to have numerous bioactivities [7,8,9,10,11,12,13,14]. For example, CNFs suppressed clinical symptoms and colon inflammation in an experimental colitis model [7,8,9]. SDACNFs suppressed increases in body weight and serum leptin level in a model of obesity induced by a high-fat diet [2] and decreased serum levels of cholesterol in a rat model of hypercholesterolemia [10]. These results indicate that CNFs and SDACNFs are potential potent functional foods for various diseases.

Metabolome analysis is used to evaluate the characteristics and interactions of low molecular weight metabolites under a specific set of conditions such as, a particular developmental stage or under certain environmental conditions [15]. The metabolome mainly represents the endpoint of the omics cascade and is the closest point to the phenotype in the cascade. Changes in metabolite levels can also be induced by exogenous factors, such as environmental and dietary factors while genomic information is not basically affected. Therefore, metabolite profiles are considered a summary of other upstream omics profiles and metabolome analysis might be able to detect subtle changes in metabolic pathways and deviations from homeostasis before phenotypic changes occur [16,17].

The gut microbiome (the collective genomes of gut microbes) encodes 3.3 million non-redundant genes, which is 150 times larger than the human gene complement [18]. This genetic richness enables the gut microbiota to perform diverse and active metabolic activities that are not encoded in the human genome, such as processing dietary polysaccharides [19,20]. In addition, the gut microbiota exchange metabolites with the host and interact with host signaling pathways to modulate functions such as host bile acid, lipid, and amino acid metabolism as well as host gene expression [21,22,23,24].

Therefore, the aim of this study was to reveal novel bioactivities of orally administered CNFs and SDACNFs. In addition, we evaluated the effects of oral administration of CNFs and SDACNFs on plasma metabolites using metabolome and gut microorganism analysis.

## 2. Results and Discussion

### 2.1. Effects of Oral Administration of CNFs and SDACNFs on Plasma Metabolites

**Figure 1:**
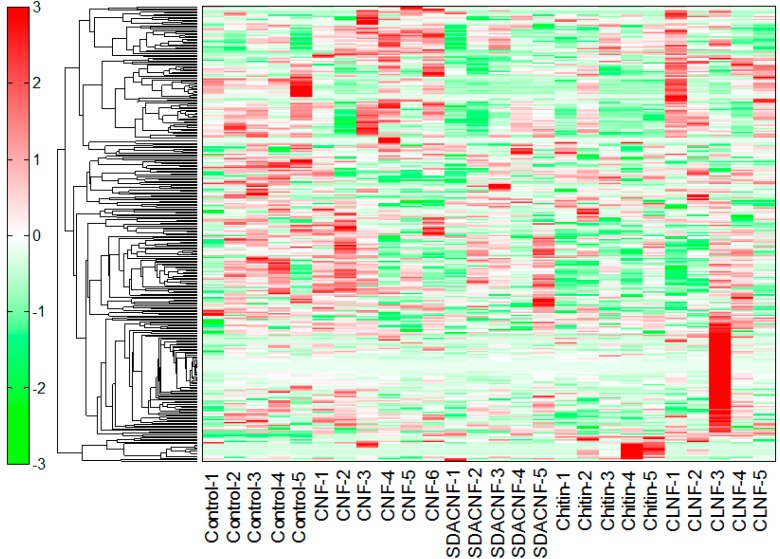
Results of hierarchical cluster analysis of plasma metabolites. An ordinate and an abscissa indicate peaks and names of experimental groups, respectively. Hierarchical cluster analysis (HCA) was performed for peaks, and distance between peaks are shown as tree diagrams. Denser green and red indicate shorter and longer distances than those of the control group, respectively. CNF, chitin nanofiber; SDACNF, surface-deacetylated CNF; CLNF, cellulose nanofiber.

In this study, capillary electrophoresis-time-of-flight mass spectrometry (CE-TOFMS) revealed 224 peaks (129 cations and 95 anions). Liquid chromatography (LC)-TOFMS detected 90 peaks (49 positive and 41 negative). The results of the hierarchical cluster analysis (HCA) are shown in Figure 1. Relative areas of metabolites after oral administration of CNFs, SDACNFs, chitin, and cellulose nanofibers (CLNFs) were compared with those of the control group. All *p*-values were determined using Welch’s *t*-test. The ratios of the metabolite concentrations to that of the control group are shown in Table 1.

**Table 1:** Ratios of relative areas of nanofiber metabolites to control group.

Compound	Comparative Analysis							
	CNF/Control		SDACNF/Control		Chitin/Control		CLNF/Control	
	Ratio	*p*-Value	Ratio	*p*-Value	Ratio	*p*-Value	Ratio	*p*-Value
ATP	2.9	0.003	1.2	0.516	1.5	0.070	1.5	0.122
2,3-Diphosphoglyceric acid	2.7	0.003	1.1	0.805	1.5	0.238	1.5	0.277
ADP	2.2	0.003	1.1	0.678	1.4	0.201	1.3	0.346
5-Hydroxytryptophan	2.0	0.003	1.8	0.151	2.1	0.065	2.0	0.051
ADP-ribose	1.8	0.003	1.2	0.429	1.1	0.689	1.3	0.447
UTP	1.7	0.028	1.2	0.161	1.0	0.986	1.4	N.A.
Cystine	1.7	0.003	1.6	0.009	3.4	1.1 × 10^−6^	2.3	5.2 × 10^−4^
Serotonin	1.6	0.014	1.4	0.238	1.6	0.056	1.3	0.240
Urocanic acid	1.6	0.058	0.8	0.013	1.0	0.750	1.2	0.202
2,6-Diaminopimelic acid	1.5	0.043	1.1	N.A.	1.7	0.040	2.4	0.253
1-Methylhistamine	1.5	0.031	1.1	0.301	1.0	0.764	1.2	0.610
2-Aminoisobutyric acid	1.4	0.023	0.9	0.470	1.0	0.723	1.3	0.041
2-Hydroxyisobutyric acid	1.4	0.017	1.0	0.618	1.1	0.208	1.2	0.353
*N*^5^-Ethylglutamine	1.3	0.041	0.9	0.566	0.8	0.104	0.7	0.080
Thiamine	1.3	0.018	1.0	0.950	0.7	0.095	0.9	0.617
Putrescine	1.2	0.002	1.0	0.707	1.1	0.469	1.3	0.305
Trimethylamine *N*-oxide	1.1	0.696	1.6	0.013	1.4	0.238	2.2	0.109
*N*-Acetylglycine	1.1	0.324	0.9	0.040	1.0	0.721	1.2	0.054
FA(17:1)	0.9	0.544	0.7	0.022	0.7	0.013	0.9	0.253
Cystathionine	0.9	0.223	0.8	0.028	1.1	0.485	0.9	0.633
Stearic acid	0.9	0.349	0.8	0.033	0.8	0.016	0.9	0.353
Glycolic acid	0.8	0.026	1.0	0.864	1.0	0.826	1.0	0.934
Glu	0.8	0.038	0.8	0.101	0.8	0.031	0.8	0.042
Betaine	0.8	0.046	0.9	0.220	1.1	0.372	0.9	0.184
Argininosuccinic acid	0.7	0.027	0.7	0.024	0.6	0.005	0.8	0.429
AC(18:0)	0.7	0.007	0.6	0.002	0.5	4.9 × 10^−4^	0.8	0.140
Palmitoylcarnitine	0.7	0.138	0.4	0.019	0.4	0.020	0.7	0.221
AC(18:1)	0.6	0.080	0.4	0.019	0.5	0.029	0.8	0.525
AC(18:2)	0.5	0.015	0.4	0.008	0.5	0.013	0.8	0.431
Taurodeoxycholic acid	0.4	0.028	0.4	0.025	0.5	0.056	0.5	0.083
20α-Hydroxyprogesterone	0.4	0.013	0.7	0.176	0.8	0.375	0.9	0.734
Taurocholic acid	0.3	0.028	0.9	0.872	0.4	0.042	0.6	0.122

All the *p*-values were determined using Welch’s *t*-test. CNF, chitin nanofiber; SDACNF, surface-deacetylated CNF; CLNF, cellulose nanofiber; ATP, adenosine triphosphate; ADP, adenosine diphosphate; UTP, uridine triphosphate; Glu, glucose; N.A., not available.

**Figure 2:**
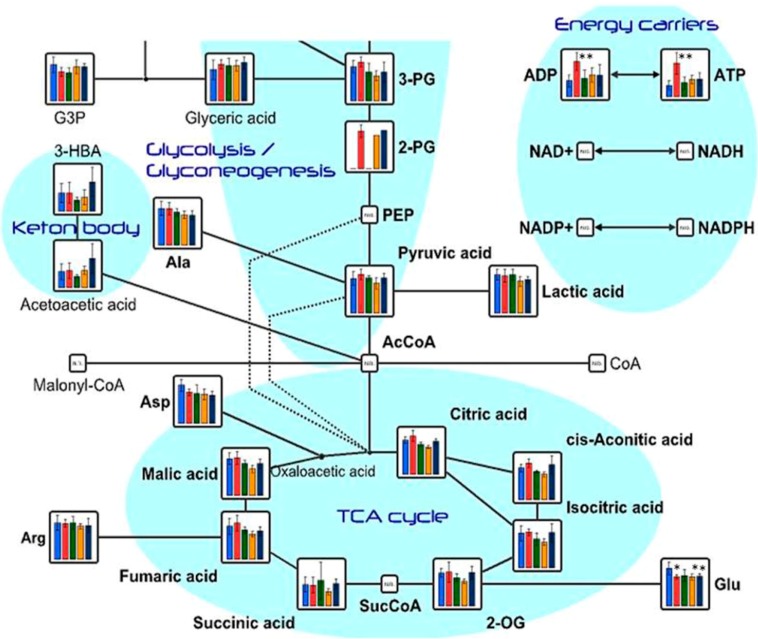
Levels of plasma metabolites related to tricarboxylic acid (TCA) cycle, nucleotides sugars, and pentose phosphate pathways. Each bar indicates median ± standard deviation (SD) of metabolite in each group. * *p* < 0.05, ** *p* < 0.01 indicate significant differences in metabolite concentrations compared to the control group. All the *p*-values were determined using Welch’s *t*-test. (blue, control; red, CNFs; green, SDACNFs; orange, chitin; navy, cellulose nanofiber (CLNF); G3P, d-glyceraldehyde 3-phosphate; 3-PG, 3-phosphoglycerate; 2-PG, 2-phosphoglycerate; ADP, adenosine diphosphate; ATP, adenosine triphosphate; 3-HBA, 3-hydroxybenzoate; PEP, phosphoenolpyruvate; NAD, nicotinamide adenine dinucleotide; NADP, nicotinamide adenine dinucleotide phosphate; NADPH, reduced nicotinamide adenine dinucleotide phosphate; Ala, alanine; Asp, aspartate; AcCoA, acetyl coenzyme A; Arg, arginine; SucCoA, succinyl coenzyme A; Glu, glucose; 2-OG, 2-oleoylglycerol).

The results of the determination of metabolite concentrations are shown in Table 2. The results of relative areas are shown in Table 3. In the CNFs group, plasma levels of adenosine triphosphate (ATP) and adenosine diphosphate (ADP) were significantly higher than they were in the control group. On the other hand, no increase in plasma levels of ATP and ADP were observed in the SDACNF, chitin, and CLNF groups. ATP is the source of energy in living cells and acts as an allosteric effector of numerous cell processes such as active transport, nucleic acid synthesis, muscle activity, and movement [25,26,27,28,29]. Endogenous ATP and ADP can be released into the extracellular milieu as a consequence of several mechanisms including cell lysis, opening channel-like pathways, and exocytosis of secretory vesicles [30,31]. In addition to their roles as intracellular energy transporters, the purine nucleotides ATP and ADP are important extracellular signaling molecules [32]. Figure 2 shows the results of plasma metabolite levels related to the tricarboxylic acid cycle (TCA) cycle. Although there was no significant difference, plasma levels of metabolites related the TCA cycle were higher in the treated groups than they were in the control group. These results might indicate that oral administration of CNFs increase plasma ATP level by affecting the TCA cycle. The results revealed that only the CNF group showed plasma serotonin (5-hydroxytryptamine, 5-HT) and 5-hydroxytryptophan (5-HTP) levels that were significantly higher than those of the control group (Table 1). Tryptophan is converted to 5-HT via 5-HTP [33]. It is well known that 5-HT act as a brain neurotransmitter. Moreover, it is an important regulatory factor in the gastrointestinal tract (GIT) and other organ systems. More than 90% of the body’s 5-HT is synthesized in the gut [34]. More recently, it was reported that some gut microbiota synthesize 5-HT [35] while some others secrete ATP [29]. The results of the present study might indicate that CNFs stimulate the functions of gut microbiota.

**Figure 3:**
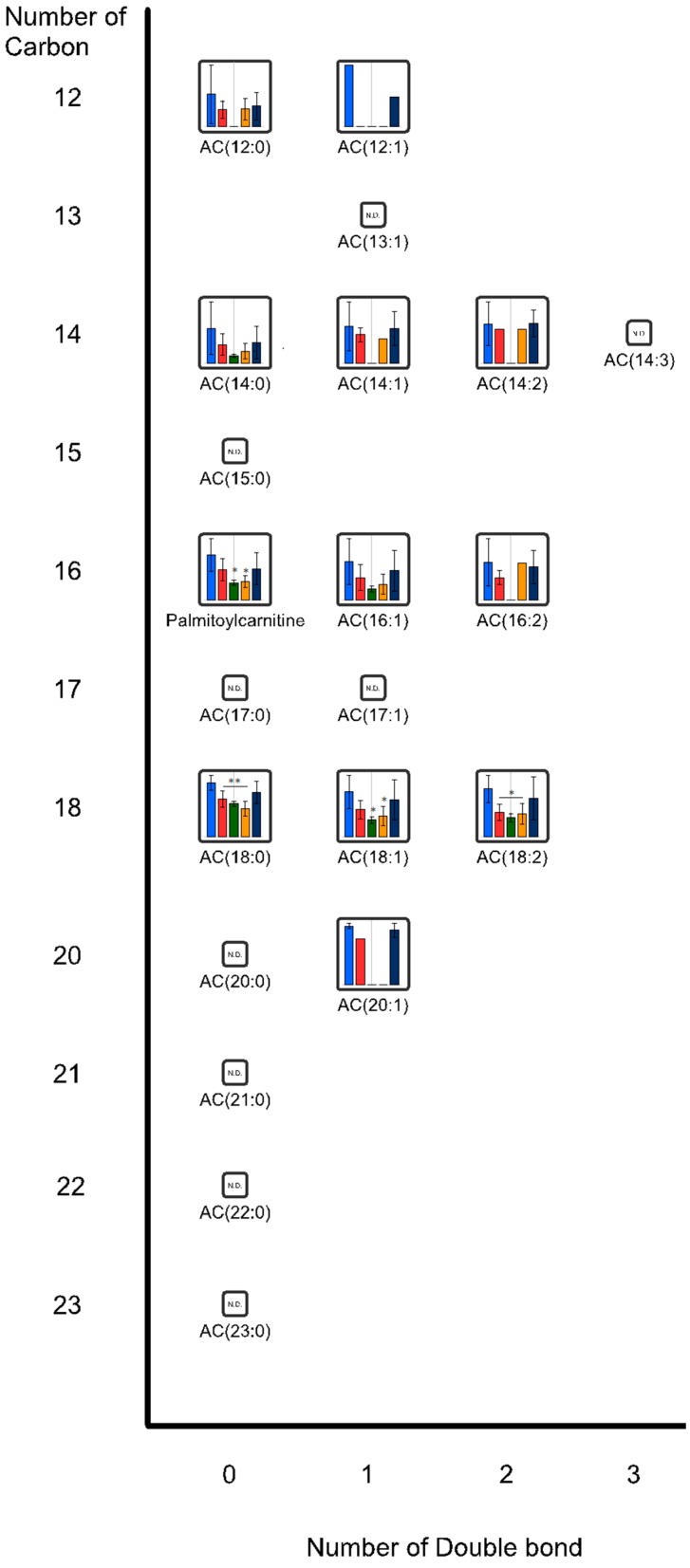
Plasma acyl-carnitine (AC) levels. Each bar represents median ± standard deviation (SD) of metabolite in each group. * *p* < 0.05, ** *p* < 0.01 indicate significant differences in metabolite concentrations compared to the control group. All the *p*-values were determined using Welch’s *t*-test. (blue, control; red, chitin nanofibers (CNFs); green, surface-deacetylated (SDA) CNFs; orange, chitin; navy, cellulose nanofiber (CLNF); N.D.: not detected).

**Figure 4:**
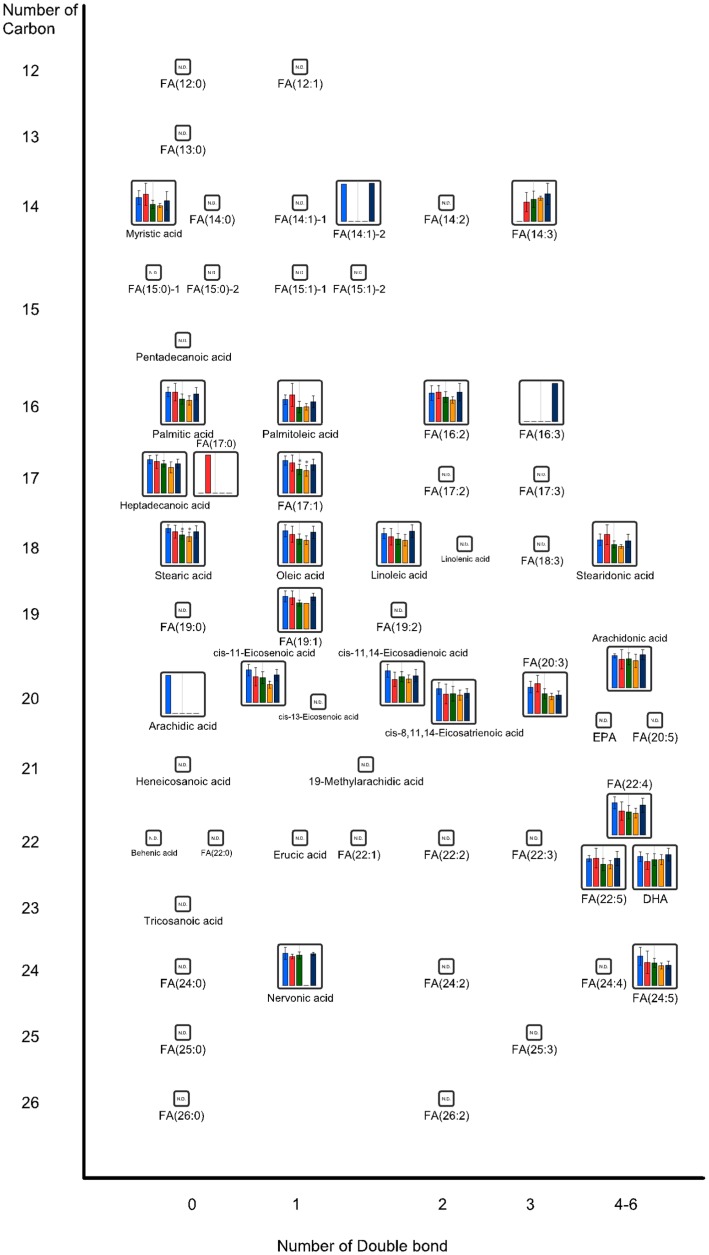
Plasma fatty acid (FA) levels. Each bar represents median ± standard deviation (SD) of metabolite in each group. * *p* < 0.05, ** *p* < 0.01 indicate significant differences in metabolite concentrations compared to the control group. All the *p*-values were determined using Welch’s *t*-test. (blue, the control; red, chitin nanofibers (CNFs); green, surface-deacetylated (SDA) CNFs; orange, chitin; navy, cellulose nanofiber (CLNF); N.D., not detected).

Figure 3 and Figure 4 show the effects of oral administration of CNFs and SDACNFs on plasma acyl-carnitine (AC) and fatty acid (FA) levels. The SDACNFs group showed plasma levels of FA(17:1), FA(22:4), AC(18:0), AC(18:1), and AC(18:2) that were significantly lower than those of the control group (Table 1). In particular, long-chain ACs perform important carrier functions during FA β-oxidation. First, carnitine palmitoyltransferase I (CPT I) catalyzes the transfer of acyl groups from acyl-coenzyme A to free carnitine to form ACs outside the mitochondrial membrane. Next, the ACs are transported to the mitochondrial matrix where the acyl groups are transferred to coenzyme A to form acyl-coenzyme A, which can participate in β-oxidation to supply energy [36]. When incomplete β-oxidation of fatty acids occurs within the mitochondria, ACs accumulate in the plasma [36]. Plasma AC concentrations have been shown to be elevated in individuals who are obese with either impaired glucose tolerance or diabetes [37,38]. Some reports described that intake of high fat diet increase blood AC levels in both experimental animals and humans [39,40,41]. It is indicated that plasma free FA levels are elevated in obese individuals [42], most likely due to increased free FA release associated with an expansion in fat mass [43,44]. In this study, significant decreases of plasma levels of FA(17:1), FA(22:4), AC(18:0), AC(18:1), and AC(18:2) were observed. The results of the present study indicate that one possible mechanism of the anti-obesity effects of orally administered SDACNFs might involve the tuning of lipid metabolism. To the best of our knowledge, there are few reports that describe the relationship between oral administration of chitin derivatives and changes in plasma ACs or FAs. Further study must be perform to this point.

Figure 3 and Figure 4 show the effects of oral administration of CNFs and SDACNFs on plasma acyl-carnitine (AC) and fatty acid (FA) levels. The SDACNFs group showed plasma levels of FA(17:1), FA(22:4), AC(18:0), AC(18:1), and AC(18:2) that were significantly lower than those of the control group (Table 1). In particular, long-chain ACs perform important carrier functions during FA β-oxidation. First, carnitine palmitoyltransferase I (CPT I) catalyzes the transfer of acyl groups from acyl-coenzyme A to free carnitine to form ACs outside the mitochondrial membrane. Next, the ACs are transported to the mitochondrial matrix where the acyl groups are transferred to coenzyme A to form acyl-coenzyme A, which can participate in β-oxidation to supply energy [36]. When incomplete β-oxidation of fatty acids occurs within the mitochondria, ACs accumulate in the plasma [36]. Plasma AC concentrations have been shown to be elevated in individuals who are obese with either impaired glucose tolerance or diabetes [37,38]. Some reports described that intake of high fat diet increase blood AC levels in both experimental animals and humans [39,40,41]. It is indicated that plasma free FA levels are elevated in obese individuals [42], most likely due to increased free FA release associated with an expansion in fat mass [43,44]. In this study, significant decreases of plasma levels of FA(17:1), FA(22:4), AC(18:0), AC(18:1), and AC(18:2) were observed. The results of the present study indicate that one possible mechanism of the anti-obesity effects of orally administered SDACNFs might involve the tuning of lipid metabolism. To the best of our knowledge, there are few reports that describe the relationship between oral administration of chitin derivatives and changes in plasma ACs or FAs. Further study must be perform to this point.

**Table 2:** Relative areas of plasma metabolite among experimental groups.

Compound Name	Control		CNF		SDACNF		Chitin		CLNF	
	Mean	SD	Mean	SD	Mean	SD	Mean	SD	Mean	SD
ATP	8.0 × 10^−4^	3.1 × 10^−4^	2.3 × 10^−3^	7.5 × 10^−4^	9.6 × 10^−4^	4.3 × 10^−4^	1.2 × 10^−3^	3.0 × 10^−4^	1.2 × 10^−3^	4.5 × 10^−4^
2,3-Diphosphoglyceric acid	6.4 × 10^−4^	1.5 × 10^−4^	1.7 × 10^−3^	5.2 × 10^−4^	6.7 × 10^−4^	2.6 × 10^−4^	9.6 × 10^−4^	5.2 × 10^−4^	9.8 × 10^−4^	5.9 × 10^−4^
ADP	7.8 × 10^−4^	3.0 × 10^−4^	1.7 × 10^−3^	4.4 × 10^−4^	8.8 × 10^−4^	4.3 × 10^−4^	1.1 × 10^−3^	3.5 × 10^−4^	1.1 × 10^−3^	5.1 × 10^−4^
5-Hydroxytryptophan	8.4 × 10^−6^	1.9 × 10^−6^	1.7 × 10^−5^	4.0 × 10^−6^	1.5 × 10^−5^	6.9 × 10^−6^	1.8 × 10^−5^	8.5 × 10^−6^	1.7 × 10^−5^	5.6 × 10^−6^
ADP-ribose	1.2 × 10^−4^	4.0 × 10^−5^	2.2 × 10^−4^	3.6 × 10^−5^	1.5 × 10^−4^	5.9 × 10^−5^	1.3 × 10^−4^	2.9 × 10^−5^	1.7 × 10^−4^	1.1 × 10^−4^
UTP	9.4 × 10^−5^	1.8 × 10^−5^	1.6 × 10^−4^	5.3 × 10^−5^	1.2 × 10^−4^	1.5 × 10^−6^	9.4 × 10^−5^	2.1 × 10^−5^	1.3 × 10^−4^	N.A.
Cystine	7.4 × 10^−4^	2.1× 10^−4^	1.2 × 10^−4^	1.2 × 10^−4^	1.2 × 10^−4^	1.4 × 10^−4^	2.5 × 10^−3^	2.1 × 10^−4^	1.7 × 10^−3^	2.9 × 10^−4^
Serotonin	1.3 × 10^−4^	4.7 × 10^−5^	2.1 × 10^−4^	3.1 × 10^−5^	1.8 × 10^−4^	7.5 × 10^−5^	2.1 × 10^−4^	6.3 × 10^−5^	1.7 × 10^−4^	5.2 × 10^−5^
Urocanic acid	2.1 × 10^−4^	2.3 × 10^−5^	3.3 × 10^−4^	1.2 × 10^−4^	1.7 × 10^−4^	1.7 × 10^−5^	2.1 × 10^−4^	3.4 × 10^−5^	2.5 × 10^−4^	6.5 × 10^−5^
32,6-Diaminopimelic acid	5.8 × 10^−5^	8.4 × 10^−6^	8.6 × 10^−5^	1.2 × 10^−5^	6.6 × 10^−5^	N.A.	9.8 × 10^−5^	8.2 × 10^−6^	1.4 × 10^−4^	8.6 × 10^−5^
1-Methylhistamine	1.2 × 10^−4^	1.1 × 10^−5^	1.8 × 10^−4^	4.8 × 10^−5^	1.4 × 10^−4^	2.4 × 10^−5^	1.2 × 10^−4^	1.6 × 10^−5^	1.4 × 10^−4^	7.8 × 10^−5^
2-Aminoisobutyric acid	2.4 × 10^−3^	5.2 × 10^−4^	3.4 × 10^−3^	6.7 × 10^−4^	2.2 × 10^−3^	4.5 × 10^−4^	2.3 × 10^−3^	3.2 × 10^−4^	3.2 × 10^−3^	4.8 × 10^−4^
2-Hydroxyisobutyric acid	5.2 × 10^−4^	9.8 × 10^−5^	7.3 × 10^−4^	1.3 × 10^−4^	5.0 × 10^−4^	5.0 × 10^−4^	6.0 × 10^−4^	7.4 × 10^−5^	6.0 × 10^−4^	1.5 × 10^−4^
*N*^5^-Ethylglutamine	1.3 × 10^−3^	3.4 × 10^−4^	1.7 × 10^−3^	2.2 × 10^−4^	1.2 × 10^−3^	2.1 × 10^−4^	9.8 × 10^−4^	1.2 × 10^−4^	9.4 × 10^−4^	1.7 × 10^−4^
Thiamine	1.2 × 10^−4^	2.1 × 10^−5^	1.6 × 10^−4^	1.6 × 10^−5^	1.3 × 10^−4^	7.9 × 10^−5^	9.3 × 10^−5^	2.9 × 10^−5^	1.2 × 10^−4^	2.9 × 10^−5^
Putrescine	1.5 × 10^−4^	1.1 × 10^−5^	1.8 × 10^−4^	1.0 × 10^−5^	1.5 × 10^−4^	2.4 × 10^−5^	1.6 × 10^−4^	1.8 × 10^−5^	1.9 × 10^−4^	7.5 × 10^−5^
Trimethylamine *N*-oxide	4.6 × 10^−3^	7.7 × 10^−4^	5.0 × 10^−3^	2.6 × 10^−3^	7.4 × 10^−3^	1.6 × 10^−3^	6.6 × 10^−3^	3.2 × 10^−3^	9.9 × 10^−3^	5.8 × 10^−3^
*N*-Acetylglycine	1.7 × 10^−4^	1.5 × 10^−5^	1.8 × 10^−4^	1.7 × 10^−5^	1.5 × 10^−4^	1.5 × 10^−5^	1.7 × 10^−4^	2.7 × 10^−5^	2.2 × 10^−4^	3.5 × 10^−5^
FA(17:1)	3.2 × 10^−5^	4.6 × 10^−6^	3.0 × 10^−5^	7.8 × 10^−6^	2.3 × 10^−5^	5.1 × 10^−6^	2.2 × 10^−5^	5.2 × 10^−6^	2.8 × 10^−5^	5.4 × 10^−6^
Ser	4.3 × 10^−2^	9.7 × 10^−3^	4.0 × 10^−2^	5.6 × 10^−3^	4.0 × 10^−2^	4.3 × 10^−3^	3.7 × 10^−2^	4.6 × 10^−3^	3.7 × 10^−2^	6.1 × 10^−3^
Cytidine	5.9 × 10^−4^	1.1 × 10^−4^	5.4 × 10^−4^	1.6 × 10^−4^	5.4 × 10^−4^	9.3 × 10^−5^	6.2 × 10^−4^	5.3 × 10^−5^	7.0 × 10^−4^	1.3 × 10^−4^
Cystathionine	2.6 × 10^−4^	2.1 × 10^−5^	2.4 × 10^−4^	2.7 × 10^−5^	2.2 × 10^−4^	2.6 × 10^−4^	2.8 × 10^−4^	5.7 × 10^−5^	2.5 × 10^−4^	4.0 × 10^−5^
Stearic acid	1.1 × 10^−3^	1.2 × 10^−4^	9.7 × 10^−4^	2.0 × 10^−4^	8.7 × 10^−4^	1.2 × 10^−4^	8.1 × 10^−4^	1.4 × 10^−4^	9.7 × 10^−4^	1.9 × 10^−4^
Glyoxylic acid	1.4 × 10^−4^	4.0 × 10^−4^	1.3 × 10^−4^	2.0 × 10^−5^	1.4 × 10^−4^	2.4 × 10^−4^	1.4 × 10^−4^	2.2 × 10^−5^	1.9 × 10^−4^	5.8 × 10^−5^
Glu	1.4 × 10^−2^	2.4 × 10^−3^	1.1 × 10^−2^	8.0 × 10^−4^	1.1 × 10^−2^	2.3 × 10^−3^	1.1 × 10^−2^	1.1 × 10^−3^	1.1 × 10^−2^	9.1 × 10^−4^
Betaine	5.1 × 10^−2^	9.3 × 10^−3^	3.9 × 10^−2^	5.9 × 10^−3^	4.4 × 10^−2^	4.7 × 10^−3^	5.6 × 10^−2^	1.0 × 10^−2^	4.4 × 10^−2^	4.5 × 10^−3^
Argininosuccinic acid	1.7 × 10^−3^	2.9 × 10^−5^	1.3 × 10^−4^	2.5 × 10^−5^	1.3 × 10^−4^	2.7 × 10^−5^	1.1 × 10^−4^	2.6 × 10^−5^	1.4 × 10^−4^	7.5 × 10^−5^
AC(18:0)	5.3 × 10^−5^	7.2 × 10^−6^	3.7 × 10^−5^	8.0 × 10^−6^	3.2 × 10^−5^	2.8 × 10^−6^	2.8 × 10^−5^	7.0 × 10^−6^	4.3 × 10^−5^	1.1 × 10^−5^
Palmitoylcarnitine	3.5 × 10^−4^	1.3 × 10^−4^	2.4 × 10^−4^	8.7 × 10^−5^	1.3 × 10^−4^	1.9 × 10^−5^	1.5 × 10^−4^	4.7 × 10^−5^	2.4 × 10^−4^	1.2 × 10^−4^
AC(18:1)	2.6 × 10^−4^	9.7 × 10^−5^	1.6 × 10^−4^	5.5 × 10^−5^	9.8 × 10^−5^	1.9 × 10^−5^	1.2 × 10^−4^	5.4 × 10^−5^	2.2 × 10^−4^	1.2 × 10^−4^
AC(18:2)	1.2 × 10^−4^	3.5 × 10^−5^	6.3 × 10^−5^	2.1 × 10^−5^	5.0 × 10^−5^	1.1 × 10^−5^	5.9 × 10^−5^	2.7 × 10^−5^	9.9 × 10^−5^	5.5 × 10^−5^
Taurodeoxycholic acid	6.2 × 10^−5^	2.5 × 10^−5^	2.5 × 10^−5^	6.2 × 10^−6^	2.5 × 10^−5^	9.1 × 10^−6^	3.1 × 10^−5^	1.7 × 10^−5^	3.4 × 10^−5^	1.9 × 10^−5^
20α-Hydroxyprogesterone	1.3 × 10^−5^	4.8 × 10^−5^	4.9 × 10^−5^	2.2 × 10^−5^	9.6 × 10^−5^	2.9 × 10^−5^	1.1 × 10^−4^	3.1 × 10^−5^	1.2 × 10^−4^	8.6 × 10^−5^
Taurocholic acid	4.0 × 10^−4^	1.8 × 10^−4^	1.4 × 10^−4^	3.1 × 10^−5^	3.7 × 10^−4^	3.3 × 10^−4^	1.7 × 10^−4^	1.1 × 10^−4^	2.4 × 10^−4^	7.6 × 10^−5^

CNF, chitin nanofiber; SDACNF, surface-deacetylated CNF; CLNF, cellulose nanofiber; ATP, adenosine triphosphate; ADP, adenosine diphosphate; UTP, uridine triphosphate; Glu, glucose; N.A., not available.

### 2.2. Effects of Oral Administrations of CNFs and SDACNFs on Gut Microbiota and Fecal Organic Acid Levels

The results of the gut microbiota analysis are shown in Table 3. The SDACNF group showed a more significant increase in the content of *Bacteroidales* than the control group did. On the other hand, no change in gut microbiota was observed in the control, CNF, chitin, and CLNF groups. The results of the determination of fecal short-chain FAs (SCFA) content are shown in Table 4. The level of fecal lactic acid was significantly increased in the CNF group compared with that of the control group. Furthermore, the level of fecal acetic acid in the CNF group was significantly higher than that of the control group was. In the SDACNF group, the level of fecal propionic acid was significantly higher than that of the control group.

Previously, it was demonstrated that gut microbiota are involved in the maturation and regulation of host immunity and gut functions [45]. The capsular antigen of the human commensal *Bacteroidales fragilis* triggers T cell-dependent immune responses that can affect both the development and homeostasis of the host immune system [46,47,48]. Some reports indicate that gut *Bacteroidales* is associated with numerous diseases including inflammatory bowel disease [49,50], rheumatoid arthritis [51], and obesity [52]. The results of this study suggest that the anti-obesity effects of orally administered SDACNFs may be induced by changes in the population of gut *Bacteroidales*.

The main products of intestinal bacterial fermentation of dietary fiber are SCFAs such as lactic acid, acetate, and propionate [53]. SCFAs can be used for de novo synthesis of lipids and glucose, which are the main energy sources of the host [54,55]. Previous reports indicate that some microbiota promote 5-HT biosynthesis from colonic enterochromaffin (EC) cells, which supply 5-HT to the mucosa, lumen, and circulating platelets [35]. Importantly, microbiota-dependent effects on gut 5-HT significantly affect the host physiology by modulating GIT motility and platelet functions. Reigsted *et al.* [56] in particular, reported that SCFAs promoted tryptophan hydroxylase 1 transcription in a human EC cell model [56]. Furthermore, their results indicate that the actions of the gut microbiota mediated by SCFAs, are an important determinant of enteric 5-HT production and homeostasis. The results of the present study strongly indicate that the increase in plasma 5-HT level might be induced by the activation of gut microbiota functions by oral administration of CNFs.

**Table 3:** Effects of oral administrations of chitin nanofibers (CNFs) and surface-deacetylated (SDA) CNFs on gut microbiota.

% Peak Area	Control	CNF	SDACNF	Chitin	CLNF
*Bacteroidales*	36.8 ± 3.5	38.1 ± 6.5	52.0 ± 6.6 *	42.3 ± 3.4	41.1 ± 5.2
*Lactobacillus*	28.9 ± 14.2	27.6 ± 5.2	18.1 ± 12.9	27.7 ± 2.8	21.4 ± 6.6
*Clostridiales*	18.5 ± 12.4	14.0 ± 3.9	15.4 ± 12.6	15.1 ± 7.0	21.4 ± 6.1
*Erysipelotrichaceae*	1.5 ± 0.7	1.6 ± 0.4	1.0 ± 0.3	2.1 ± 0.7	2.1 ± 0.5
*Akkermansia*	0.3 ± 0.6	0.0 ± 0.0	0.0 ± 0.0	0.1 ± 0.2	0.0 ± 0.0
*Anaeroplasma*	0.3 ± 0.2	0.4 ± 0.7	0.1 ± 0.1	0.1 ± 0.1	0.0 ± 0.0
*Corinebacteriales*	0.6 ± 0.4	0.5 ± 0.1	0.5 ± 0.4	0.5 ± 0.3	0.7 ± 0.2
*Mucispirillum*	2.5 ± 1.7	1.7 ± 0.2	1.4 ± 1.2	2.4 ± 0.3	1.3 ± 0.4
*Parasutterella*	2.1 ± 1.5	4.1 ± 1.3	1.9 ± 1.0	2.0 ± 0.8	3.2 ± 1.2

Data are mean ± standard deviation (SD), * *p* < 0.05 compared to control group.

**Table 4:** Effects of oral administrations of chitin nanofibers (CNFs) and surface-deacetylated (SDA) CNFs on fecal short chain fatty acid (FA) levels.

mg/g	Control	CNF	SDACNF	Chitin	CLNF
Lactic acid	2.1 ± 0.8	4.2 ± 1.2 *	1.6 ± 1.3	1.6 ± 0.5	2.3 ± 0.6
Acetic acid	3.3 ± 0.7	4.1 ± 0.7 ^†^	3.8 ± 0.8	3.3 ± 0.4	2.7 ± 0.3
Propionic acid	0.3 ± 0.1	0.4 ± 0.1	0.5 ± 0.2 *	0.5 ± 0.1	0.3 ± 0.1
n-Butyric acid	0.1 ± 0.1	0.2 ± 0.1	0.2 ± 0.1	0.2 ± 0.1	0.2 ± 0.1

Data are mean ± standard deviation (SD), * *p* < 0.05 and ^†^ *p* < 0.05 compared to control and cellulose nanofiber (CLNF) groups, respectively.

## 3. Experimental Section

### 3.1. Animals and Reagents

Female BALB/c mice (five-week-old) were purchased from CLEA Japan (Osaka, Japan). The animals were maintained under standard conditions and allowed to acclimate for seven days before being used in the experiments. The experiments involving the mice including the procedures performed, were approved by the Animal Research Committee of Tottori University (Tottori, Japan). All experiments were performed as per ethical guidelines of Tottori University (Tottori, Japan).

Chitin powder from crab shells (chitin TC-L) was purchased from Koyo Chemical. Co., Ltd. (Tokyo, Japan). The CLNF (biomass nanofiber, BiNFi-s cellulose, WMa-10002) was purchased from Sugino Macine Co., Ltd. (Uodu, Japan). Concentrations of all samples used in this study were at 1.0 wt %.

### 3.2. Preparations of CNF, SDACNF, and CLNF

CNFs were prepared following a previously reported procedure [3]. Chitin (2.0 g) was dispersed in 200 mL of distilled water and roughly crushed with a grinder (MKCA6-2, Masuko Sanyo Co., Ltd., Kawaguchi, Japan) at clearance: −1.5 (−1.5 mm), rotating speed: 1500 rpm. The crushed dispersion was passed through a Star Burst Mini system (HJP-25001S, Sugino Machine Co., Ltd., Uodu, Japan) equipped with a ball collision chamber. The slurry was ejected through a small nozzle with a diameter of 100 μm under a high pressure of 200 MPa and allowed to collide with a ceramic ball with a diameter of 12.7 mm. Then, the suspension passed through 30 mechanical treatments. Commercial chitin powder and the nanofibers obtained here have a few amino groups at the C2 position with approximately 5% deacetylation. The average width of the nanofibers calculated from SPM images was 6.0 nm.

The SDACNFs were prepared using a previously reported procedure with modifications [6]. Chitin powder (40.0 g) was treated with 20% (*w*/*w*) sodium hydroxide (NaOH, 3.0 L) for 6 h under reflux and an argon atmosphere. After deacetylation, the supernatant was decanted, and the precipitate was thoroughly washed with distilled water and 0.5 wt % aqueous acetic acid by centrifugation to remove the water-soluble products of NaOH, AcONa, and alkaline hydrolyzed chitin. For mechanical disintegration, the deacetylated chitin was dispersed in 4.0 L aqueous acetic acid and then passed through a grinder (MKCA6-2) at clearance: −1.5 (−1.5 mm), rotating speed: 1500 rpm. The concentration, yield, and degree of deacetylation of the SDACNFs were 0.71, 74, and 20 wt %, respectively.

### 3.3. Study Design

The mice were randomized into five groups and administered tap water (control) and CNFs, SDACNFs, chitin, and CLNF each at a concentration of 0.1% dissolved in tap water. All the mice were fed a normal diet (CE-2; Japan CLEA, Osaka, Japan) for 28 days (from day 0–28). After a 12-h fast, blood samples were collected on day 29 in tubes on ice. The final concentration was 0.13% ethylenediaminetetraacetic acid dipotassium salt dihydrate (EDTA-2K), and the tubes were centrifuged at 1700× *g* at 4 °C for 10 min. Then, the supernatants were collected as plasma and stored in other tubes at −80 °C until the metabolome analysis.

### 3.4. Measurement of Metabolites

#### 3.4.1. CE-TOFMS Measurement

The CE-TOFMS measurements were carried out at a facility service at the Human Metabolome Technology Inc. (Tsuruoka, Japan). The plasma samples (50 μL) were added to 450 μL of methanol containing internal standards (IS, solution ID: H3304-1002, Human Metabolome Technologies, Tsuruoka, Japan) at 0 °C to inactivate the enzymes. The extract solution was then thoroughly mixed with 500 and 200 μL of chloroform and Milli-Q water, respectively and centrifuged at 2300*×* *g* and 4 °C for 5 min. The 350-μL upper aqueous layer was centrifugally filtered through a Millipore 5-kDa cutoff filter to remove the proteins, centrifugally concentrated, and then resuspended in 50 μL of Milli-Q water for the CE-MS analysis.

The CE-TOFMS measurement was carried out using an Agilent CE Capillary Electrophoresis System equipped with an Agilent 6210 Time of Flight mass spectrometer, Agilent 1100 isocratic high-performance liquid chromatography (HPLC) pump, Agilent G1603A CE-MS adapter kit, and Agilent G1607A CE-ESI-MS sprayer kit (Agilent Technologies, Waldbronn, Germany). The systems were controlled using the Agilent G2201AA ChemStation software version B.03.01 for CE (Agilent Technologies, Waldbronn, Germany). The metabolites were analyzed using a fused silica capillary (50 μm i.d. × 80 cm total length) with commercial electrophoresis buffers (solution IDs: H3301-1001 and H3302-1021 for cation and anion analysis, respectively, Human Metabolome Technologies, Tsuruoka, Japan) as the electrolyte. The samples were injected at a pressure of 50 mbar for 10 and 25 s (approximately 10 and 25 nL) for cation and anion analysis, respectively. The spectrometer was scanned from *m*/*z* 50 to 1000, and other conditions were as described previously [57,58,59].

#### 3.4.2. LC-TOFMS Measurement

The metabolome measurements were carried out at using the facility service at the Human Metabolome Technology Inc., (Tsuruoka, Japan). The plasma (or serum) samples (500 μL) were added to 1500 μL of 1% formic acid/acetonitrile containing the IS solution (solution ID: H3304-1002, Human Metabolome Technologies, Tsuruoka, Japan) at 0 °C to inactivate the enzymes. The solution was thoroughly mixed and centrifuged at 2300× *g* at 4 °C for 5 min, and the supernatant was filtrated using a Hybrid SPE phospholipid 55261-U (Supelco, Bellefonte, PA, USA) to remove phospholipids. The filtrate was then desiccated and dissolved in 100 μL of isopropanol/Milli-Q water for the LC-MS analysis.

The LC-TOFMS measurement was carried out using an Agilent LC System (Agilent 1200 series RRLC system SL) equipped with an Agilent 6230 Time of Flight mass spectrometer (Agilent Technologies, Waldbronn, Germany). The systems were controlled using the Agilent G2201AA ChemStation software version B.03.01 for CE (Agilent Technologies, Waldbronn, Germany). The cationic and anionic compounds were measured using ODS column (2 × 50 mm, 2 μm) according to methods described previously [60].

Peaks were extracted using an automatic integration software MasterHands (Keio University, Tsuruoka, Japan) to obtain peak information including *m*/*z*; migration time (MT) and retention time (RT) for the CE-TOFMS and LC-TOFMS measurements, respectively; and peak area [61]. Signal peaks corresponding to isotopomers, adduct ions, and other product ions of known metabolites were excluded, and the remaining peaks were annotated with putative metabolites from the HMT metabolite database based on their MTs/RTs and *m*/*z* values determined by the TOFMS. The tolerance range for the peak annotation was configured at ±0.5 min for MT and ±10 ppm for *m*/*z*. In addition, peak areas were normalized to those of the IS, and then the resultant relative area values were further normalized by sample amount. The HCA was performed using our proprietary software, PeakStat and SampleStat. Detected metabolites were plotted on metabolic pathway maps using the visualization and analysis of networks containing experimental data (VANTED) software [62].

### 3.5. Measurement of Gut Microbiota

The measurements of the gut microbiota were conducted using a facility service at the Central Institute for Experimental Animals (Kawasaki, Japan). The microbiota were analyzed using polymerase chain reaction (PCR) amplification and terminal-restriction fragment length polymorphism (T-RFLP) analysis. DNA was isolated from the cecal contents as follows [63]. The cecal contents were suspended in a solution containing 4 M guanidium thiocyanate, 100 mM Tris-HCl (pH 9.0), and 40 mM EDTA (pH 8.0), and then lysed in Lysing Matrix E (MP Biomedicals, Santa Ana, CA, USA) using a FastPrep-24 (MP Biomedicals, Santa Ana, CA, USA) homogenizer. Then, DNA was extracted using the phenol-chloroform method and purified with the Gel/PCRTM DNA isolation system (Viogene, New Taipei City, Taiwan). Purified DNA was amplified using a TaKaRa PCR Thermal Cycler Dice (Takara Bio, Shiga, Japan) and a set of universal primers including 5′6-FAM-labeled 341f (5′-CCTACGGGWGGCAGCAG-3′) corresponding to nucleotides 340–356 and 516r (5′-ATMACCGCGGCTGCTGG-3′) corresponding to nucleotides 517–533 of the 16S rRNA gene of *Escherichia* *coli* (accession No. J01859). The PCR was performed in a reaction mixture containing 1× Taq buffer, 200 μM dNTPs, 3 mM magnesium chloride (MgCl_2_), 0.2 μM each of the forward and reverse primers, 10 ng purified DNA, and 1 U HotStar Taq DNA polymerase (Qiagen, Venlo, The Netherlands). PCR amplification program included preheating at 94 °C for 15 min, followed by 25 cycles consisting of 94 °C for 30 s, 50 °C for 30 s, and 72 °C for 60 s followed by a final extension at 72 °C for 10 min. Amplified DNA was verified using 3% agarose gel electrophoresis. PCR products were purified using the Gel/PCRTM DNA isolation system (Viogene). The T-RFLP analysis was performed as previously described [64,65] with some modifications. Purified PCR products were digested with HpyCH4III (New England Biolabs, Ipswich, MA, USA) at 37 °C for 1 h. The lengths of the terminal restriction fragments (T-RFs) were determined using the standard size marker 1200 LIZ using an ABI PRISM 310 Genetic Analyzer and GeneScan software all from Applied Biosystems (Carlsbad, CA, USA). The lengths of the T-RFs were treated as an operational taxonomic unit (OTU) based on the 16S rDNA sequences obtained from microbiota from the rat gut. A peak OTU area was identified from the peaks detected to calculate the area under each peak. The OTU was then used to estimate the phylogenetic group using the database of the rat gut microbiota.

### 3.6. Measurements of Fecal SCFAs

The measurements of fecal SCFAs were carried out using a facility service at the Central Institute for Experimental Animals (Kawasaki, Japan). The fecal extracts were analyzed using an organic acids analysis system (Shimadzu, Kyoto, Japan) with the following conditions: column, Shim-pack SCR-102 (H) 300 mm × 8 mm (Shimadzu, Kyoto, Japan); elution, 5 mmol/L *p*-toluenesulfonic acid; reaction solution, 5 mmol/L *p*-toluenesulfonic acid with 100 μmol/L EDTA and 20 mmol/L bis-Tris; flow rate, 0.8 mL/min; oven temperature, 45 °C; analysis time, 60 min; and detection, CCD-10A (Shimadzu, Kyoto, Japan).

### 3.7. Statistical Analyses

Statistical analyses were performed using the Welch’s *t*-test. The data are presented as the mean ± standard deviation (SD) and *p* < 0.05 was considered statistically significant.

## 4. Conclusions

In the present study, we demonstrated that oral administration of CNFs increased plasma levels of ATP, ADP, and 5-HT while oral administrations of SDACNFs affected the metabolism of ACs and FAs. Furthermore, the analysis of the fecal organic levels indicated that oral administration of CNFs stimulated and activated the functions of the gut microbiota. These results indicate that oral administration of CNF increases the plasma levels of ATP and 5-HT by activating the gut microbiota.
